# ﻿Two new species of the bamboo-feeding planthopper genus *Neobelocera* Ding & Yang from China (Hemiptera, Fulgoromorpha, Delphacidae)

**DOI:** 10.3897/zookeys.1183.101123

**Published:** 2023-11-09

**Authors:** Hong-Xing Li, Xiang-Sheng Chen, Lin Yang

**Affiliations:** 1 Department of Light Industry & Chemical Engineering of Guizhou Light Industrial Technical College, Guiyang, Guizhou, 550025, China; 2 Institute of Entomology, Guizhou University, Guiyang, Guizhou, 550025, China; 3 The Provincial Special Key Laboratory for Development and Utilization of Insect Resources, Guizhou University, Guiyang, Guizhou, 550025, China

**Keywords:** Fulgoromorpha, identification key, morphology, oriental region, taxonomy

## Abstract

Two new species of the bamboo-feeding genus *Neobelocera* Ding & Yang, 1986, *N.furcata***sp. nov.** and *N.parvula***sp. nov.**, are described and illustrated from China. A key based on the male genitalia is given to distinguish species of this genus and a map provided to show their geographic distribution. Habitus photos for adults and illustrations of male genitalia are also given.

## ﻿Introduction

*Neobelocera* is an Oriental bamboo-feeding planthopper genus, belonging to tribe Tropidocephalini under subfamily Delphacinae (Hemiptera, Fulgoroidea, Delphacidae). It was established by Ding and Yang (Ding et al. 1986) with type species *Neobeloceraasymmetrica* Ding & Yang, 1986. This genus is only known to occur in southern China. Species in the genus exhibit morphological diversity in male genitalia. Detailed generic characteristics and a key for the identification of species in the genus were provided by Chen and Liang (2005). Subsequently, the genus and three species, *N.asymmetrica*, *N.zhejiangensis* (Zhu, 1988) and *N.hanyinensis* Qin & Yuan, 1998, were redescribed in a monograph on Delphacidae of China (Ding 2006). Hou and Chen (2010) revised the genus again and increased it to six species. Hu and Ding (2014) described a new species from Tibet, China. Recently, Li et al. (2020) updated the identification key of this genus and added two new species from southwest China, which led to total of nine species, viz., *N.asymmetrica* Ding & Yang, 1986, *N.zhejiangensis* (Zhu, 1988), *N.hanyinensis* Qin & Yuan, 1998, *N.lanpingensis* Chen, 2003, *N.laterospina* Chen & Liang, 2005, *N.lii* Hou & Chen, 2010, *N.medogensis* Hu & Ding, 2014, *N.biprocessa* Li, Yang & Chen, 2020 and *N.russa* Li, Yang & Chen, 2020.

Currently the tribe Tropidocephalini includes 201 species in 37 genera, of which 113 species in 23 genera occur in China (Ren et al. 2014; Bourgoin 2023). Of the Chinese genera of the tribe, *Belocera* Muir, 1913 and *Neobelocera*, have the antennae flattened, sagittate or subsagittate. A comparison of *Neobelocera* and *Belocera* shows that species in these genera look rather similar, but the two genera can be easily distinguished by the following characters: first segment of antennae with the apex unequally bifurcate, ventral apical angle much longer than dorsal apical angle, with median longitudinal carina (in *Belocera*, apex of first segment of antennae equally bifurcate, ventral apical angle subequal to dorsal apical angle, without median longitudinal carina); postclypeus in profile, apical part of median carina roundly bent (in *Belocera*, postclypeus in profile, apical part of median carina and lateral carinae sharply bent); rostrum very short, only reaching mesotrochanters (rostrum surpassing mesotrochanters in *Belocera*); and the surface of the forewing often has blackish-brown markings, in the dark portion veins bear white or yellowish-white spots (in *Belocera*, forewing often with a fuscous central longitudinal fascia, costal area light yellowish white).

Herein, two new species of *Neobelocera*, *N.furcata* sp. nov. and *N.parvula* sp. nov., are described and illustrated from Guizhou and Yunnan provinces, China. A key for identifying the species is provided and a map showing the geographic distribution of the species is also given.

## ﻿Material and methods

The morphological terminology follows Yang and Yang (1986). Dry male specimens were used for the description and illustration. External morphology was observed under a stereoscopic microscope and characters were measured with an ocular micrometer. Color pictures for the adult habitus were obtained using the KEYENCE VHX-6000 system. The genital segments of the examined specimens were macerated in 10% KOH and drawn from preparations in glycerin jelly using a Leica MZ 12.5 stereo microscope. Illustrations were scanned with a Canon CanoScan LiDE 200 and imported into Adobe Photoshop 6.0 for labeling and plate composition.

The type specimens of the new species are deposited in the Institute of Entomology, Guizhou University, Guiyang, China (GUGC).

## ﻿Taxonomy

### ﻿Genus Neobelocera

Taxon classificationAnimaliaHemipteraDelphacidae

Ding & Yang, 1986

A8EAF6E7-9D35-5BD8-8A24-CAA120051091

Figs 1–6 , 7–16 , 17–22 , 23–31

Neobelocera Ding & Yang, in Ding et al. 1986: 420; Chen and Liang 2005: 374; Ding 2006: 196; Hou and Chen 2010: 40; Li et al. 2020: 3.

#### Type species.

*Neobeloceraasymmetrica* Ding & Yang, 1986.

#### Diagnosis.

*Neobelocera* can be distinguished from other related genera of Tropidocephalini by the following characters: antennae with first segment subsagittate, markedly flattened, a longitudinal carina down middle, the ventral apical angle longer than dorsal apical angle (Figs 5, 8, 21, 24); when postclypeus viewed in profile, apical part of median carina roundly bent (Figs 4, 20); rostrum very short, only reaching mesotrochanters (Ding et al. 1986; Chen 2003; Chen and Liang 2005; Hou and Chen 2010; Hu and Ding 2014; Li et al. 2020).

**Figures 1–6.**
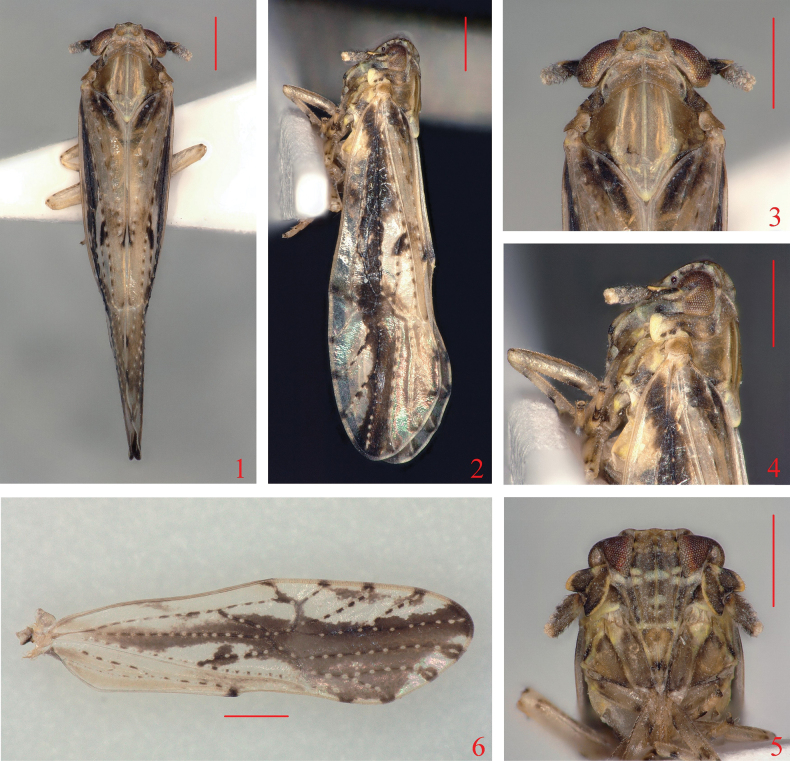
*Neobelocerafurcata* sp. nov. **1** male adult, dorsal view **2** same, lateral view **3** head and thorax, dorsal view **4** same, lateral view **5** face **6** forewing. Scale bars: 0.5 mm (**1–6**).

#### Host plant.

Bamboo.

#### Distribution.

Oriental region (China).

### ﻿Key to species (males) of *Neobelocera* Ding & Yang, 1986 (revised from Li et al. 2020)

**Table:** 

1	Forewings yellowish white, hyaline, with a small dark-brown markings on furcation of ScP (Hu and Ding 2014: fig. 10)	***N.medogensis* Hu & Ding, 2014**
–	Forewings with blackish-brown markings, with veins with white spots or white short stripes at intervals (Figs 6, 22)	**2**
2	Frons with pale transverse band below level of lower margin of eyes (Figs 5, 21)	**3**
–	Frons without transverse band (Chen and Liang 2005: fig. 10)	**8**
3	Ventral margin of pygofer with medioventral process (Figs 12, 25)	**4**
–	Ventral margin of pygofer concave medially, without process	**7**
4	Anal segment (Fig. 27) with a long ventral process medially	***N.parvula* sp. nov.**
–	Anal segment (Fig. 9) without process	**5**
5	Genital styles with apex forked (Figs 13, 14)	***N.furcata* sp. nov.**
–	Genital styles with apex not forked	**6**
6	Frons with some short, yellowish-white transverse stripes subapically; genae with 2 or 3 light brown spots (Li et al. 2020: fig. 3E); pygofer with medioventral processes short, median one slightly longer than lateral ones (Li et al. 2020: fig. 4D)	***N.russa* Li, Yang & Chen, 2020**
–	Frons without yellowish-white transverse stripe subapically; genae without light brown spot (Chen 2003: fig. 3); medioventral processes of pygofer with median one short, lateral ones very slender and long (Chen 2003: fig. 4)	***N.lanpingensis* Chen, 2003**
7	Phallus with basal half broad, compressed, apical half slender, tubular, acute at apex, with process at basal ⅓ and node subapically; phyllobase with long straight spinous process basally and three processes apically (Li et al. 2020: fig. 2G)	***N.biprocessa* Li, Yang & Chen, 2020**
–	Phallus slender, tubular, rounded at apex, without process and node; phyllobase slender, without processes (Ding et al. 1986: figs 6–4)	***N.asymmetrica* Ding & Yang, 1986**
8	Median carina of vertex, pronotum, mesonotum and frons white bordered, dark brown to blackish brown (Chen and Liang 2005: figs 9, 10)	**9**
–	Not as above, forewings at basal part and hind margin of apical part with blackish-brown markings (Hou and Chen 2010: fig. 19); ventral margin of pygofer concave medially, lateral side of which each with a short process (Hou and Chen 2010: fig. 24)	***N.lii* Hou & Chen, 2010**
9	Pygofer with ventral margin concave medially, on lateral side each with a long, slender process (Chen and Liang 2005: fig. 12); genital styles slender and long, with a spine-like process subapically (Chen and Liang 2005: figs 15, 16)	***N.laterospina* Chen, 2003**
–	Pygofer with ventral margin without any process (Qin and Yuan 1998: fig. 1D)	**10**
10	Genital styles long, parallel and slightly sinuate, with inner apical angle acute, without processes (Ding 2006: fig. 100C)	***N.zhejiangensis* (Zhu, 1988)**
–	Genital styles rather robust, apex acute, with branch lateral process terminating with 3–5 spinose processes (Qin and Yuan 1998: fig. 1F–G)	***N.hanyinensis* Qin & Yuan, 1998**

### ﻿ Neobelocera furcata sp. nov.

Taxon classificationAnimaliaHemipteraDelphacidae

E0DE211E-9CA2-5F75-B0D0-6EB81107AE26

https://zoobank.org/319D0F23-29CB-499D-B03E-D216DC93BA42

Figs 1–6 , 7–16

#### Type materials.

***Holotype*** ♂, China: Guizhou, Wengan County (26°985'N, 107°646'E), on bamboo, 5 Aug. 2020, S.S. Lv leg.; ***paratypes***, 1♂, 1♀, same data as holotype.

#### Etymology.

The species epithet is derived from the Latin word ‘*furcata*’, referring to the genital styles forked at the apex. It is a feminine in gender.

#### Measurements.

Body length including forewing: male 3.8–3.9 mm (*N* = 2), female 4.0 mm (*N* = 1).

#### Diagnosis.

Forewings (Fig. 6) with blackish-brown markings, of which veins with white spots or white short stripes at intervals. Frons (Fig. 5) with pale transverse band below level of lower margin of eyes. Ventral margin of pygofer (Fig. 12) with 3 medioventral processes, lateral ones short, tapering, median one forked at apex, with 4 processes on each side and the middle two much smaller. Anal segment (Fig. 9) without process. Genital styles (Figs 13, 14) with apex forked, outer angle about twice as long as inner angle.

**Figures 7–16.**
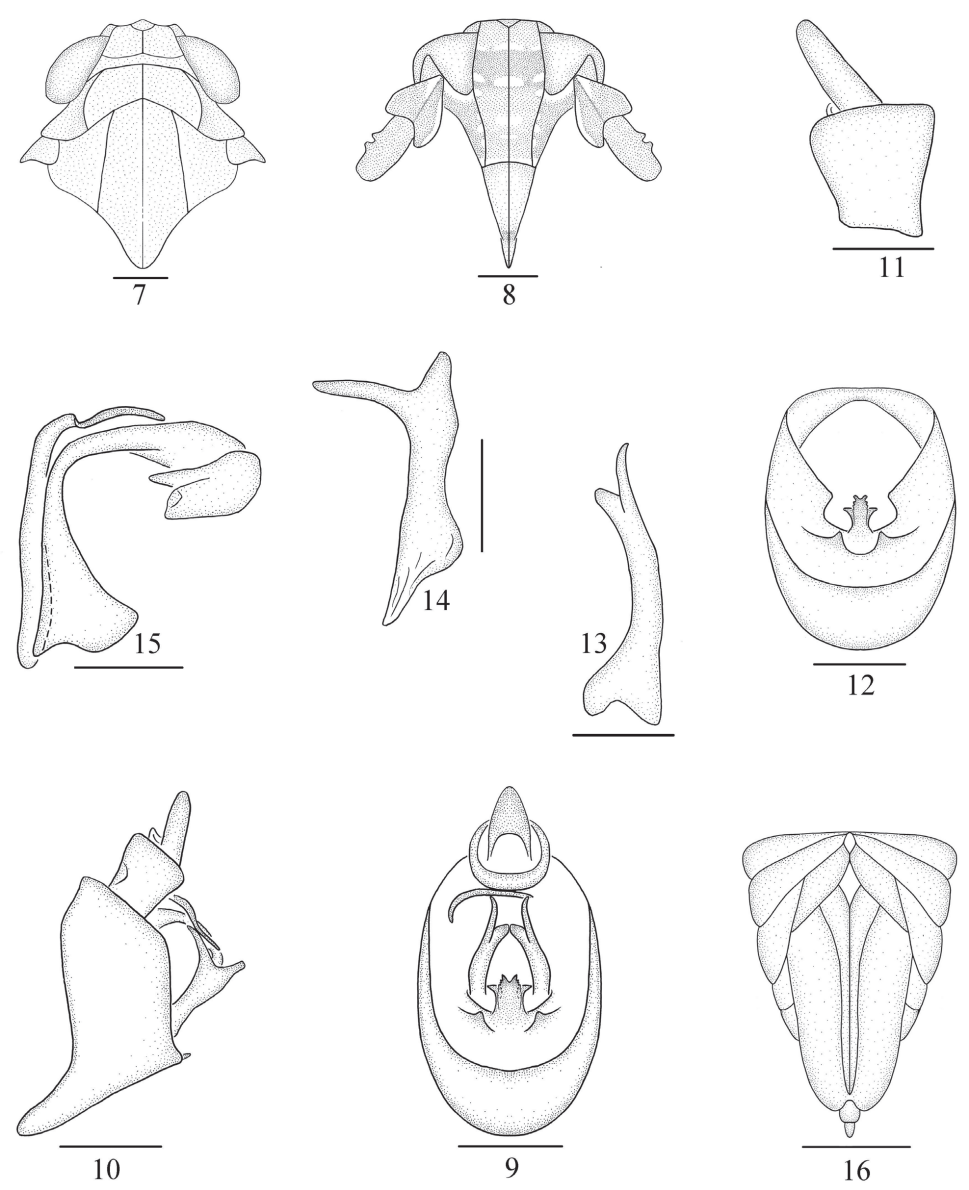
*Neobelocerafurcata* sp. nov. **7** head and thorax, dorsal view **8** face **9** male genitalia, posterior view **10** same, lateral view **11** anal segment, lateral view **12** pygofer, posterior view **13** genital style, posterior view **14** same, lateral view **15** aedeagus, lateral view **16** female genitalia, posterior view. Scale bars: 0.5 mm (**16**); 0.2 mm (**7–10, 12**); 0.1 mm (**11, 13–15**).

#### Description.

***Coloration*.** General coloration yellowish brown to dark brown (Figs 1–6). Vertex, pronotum, mesonotum (Fig. 3) dirty yellowish brown, with apex of scutellum yellowish white. Frons, genae and clypeus (Fig. 5) yellowish brown to dark brown, except broad transversal stripe below level of lower margin of eyes and narrow stripe on apex of frons yellowish white, near apex of median carina of frons and inner margin of lateral carinae of genae with several short transversal stripes yellowish white. Eyes and ocelli (Figs 4, 5) reddish brown. Antennae (Figs 3–5) yellowish brown to dark brown, except lateral margins of first segment and apex of second segment yellowish white. Legs (Fig. 5) yellowish white, with dark brown maculations. Forewing (Fig. 6) almost hyaline, along MP vein to apex with dark-brown markings, veins dark brown, with white spots at intervals. Wings hyaline, with veins dark brown. Abdomen yellowish brown to dark brown.

***Head and thorax*.** Head including eyes slightly narrower than pronotum, in profile obtusely rounded into frons (Figs 3, 4). Vertex (Figs 3, 7) broad transversely, wider at base than long medially about 2.36: 1, width at apex narrower than at base about 1: 1.84, anterior margin produced medially, Y-shaped carina distinct. Frons (Figs 5, 8) in mid line longer than wide, at widest part about 1.93: 1, widest above level of lower margin of eyes, median carina forked at extreme base. Postclypeus (Figs 5, 8) wider at base than frons at apex. Antennae (Figs 5, 8) reaching median part of postclypeus, basal segment shorter at midline than second segment about 1: 1.35, second segment long oval, somewhat compressed, longer than wide about 2.28: 1. Pronotum (Figs 3, 7) tricarinate, with anterior margin truncate, posterior margin incised strongly, lateral carinae running near anterolateral margin, then curving inward and reaching hind margin. Mesonotum (Figs 3, 7) tricarinate, longer in midline than vertex and pronotum together about 2.16: 1, median carina reaching end of scutellum. Forewing (Fig. 6) elongate, much longer than abdomen, longer in mid line than wide at widest part about 3.47: 1, predominately clear with distinctive white markings, wing apex acutely rounded; Sc, RA and RP unbranched; MP branched near wing apex, CuA 3-branched; junction of PCu + AA near mid length of clavus; fork of MP+CuA at near 2/3 length of clavus; fork RA+SC and RP near claval apex.

***Male genitalia.*** Anal segment (Fig. 9) small, ring-like, without process. Pygofer (Figs 9, 10, 12) in profile much longer ventrally than dorsally, in posterior view with opening longer than wide, ventral margin with 3 medioventral processes, lateral ones short, tapering, median one forked at apex, with 4 processes on each side and the middle two much smaller. Genital styles (Figs 13, 14) moderately long, forked at apex, outer angle about twice as long as inner angle. Aedeagus (Fig. 15) with phyllobase, phallus tubular, long, expanded at base, bent ventrad medially, broad and forked at apex, curved sharply to the left apically. Phyllobase slender, tubular, arising from base of aedeagus, running dorsad, then curving caudad, after median part, turned left then ventrad, tapering apically.

***Female genitalia*.** Female pygofer (Fig. 16) with gonocoxae broad and large, basal angle sharply acute. Ovipositor distinctly shorter than pygofer. Gonangulum broad and large, apex round with medial margin concave, connected at base to gonapophyses and gonocoxae.

#### Host plant.

Bambusoideae.

#### Distribution.

China (Guizhou).

#### Remarks.

This new species is similar to *N.russa* Li, Yang & Chen, 2020, but can be distinguished from the latter by the following features: (1) forewing (Fig. 6) along MP vein to apex with dark-brown markings [forewing with apical part from transverse veins to apex with dark-brown markings in *N.russa* (Li et al. 2020: fig. 3F)]; (2) medioventral processes of pygofer (Fig. 12) with median one forked at apex, with 4 processes on each side and the middle two much smaller [medioventral processes with median one not forked at apex, without process on each side in *N.russa* pygofer (Li et al. 2020: fig. 4D)]; and (3) apex of phallus (Fig. 15) with two processes [apex of phallus with four processes in *N.russa* (Li et al. 2020: fig. 4G)].

### ﻿ Neobelocera parvula sp. nov.

Taxon classificationAnimaliaHemipteraDelphacidae

C1BEABF2-08E8-54D6-8924-CB6551768C6B

https://zoobank.org/721F64BE-8FE0-4551-AC5F-88AF17A6CF9E

Figs 17–22 , 23–31

#### Type material.

***Holotype***: ♂, **China**: Yunnan, Jinghong County (21°586'N, 100°686'E), 19 Apr. 2020; H.X. Li leg.; **paratypes**, 8♂♂, 5♀♀, same data as holotype.

#### Etymology.

The species epithet is derived from the Latin word ‘*parvula*’, referring to the small body. It is a feminine in gender.

#### Measurements.

Body length including forewing: male 2.9–3.1 mm (*N* = 8), female 2.9–3.3 mm (*N* = 5).

#### Diagnosis.

Forewings (Fig. 22) with blackish-brown markings, of which veins with white spots or white short stripes at intervals. Frons (Fig. 21) with pale transverse band below level of lower margin of eyes. Ventral margin of pygofer (Fig. 25) with medioventral process, three branched medially, right branch much longer than the other two, sinuate, median branch nearly equal to left branch, left branch with a small process near apex. Anal segment (Fig. 27) with a long ventral process medially, bent ventrad medially.

**Figures 17–22.**
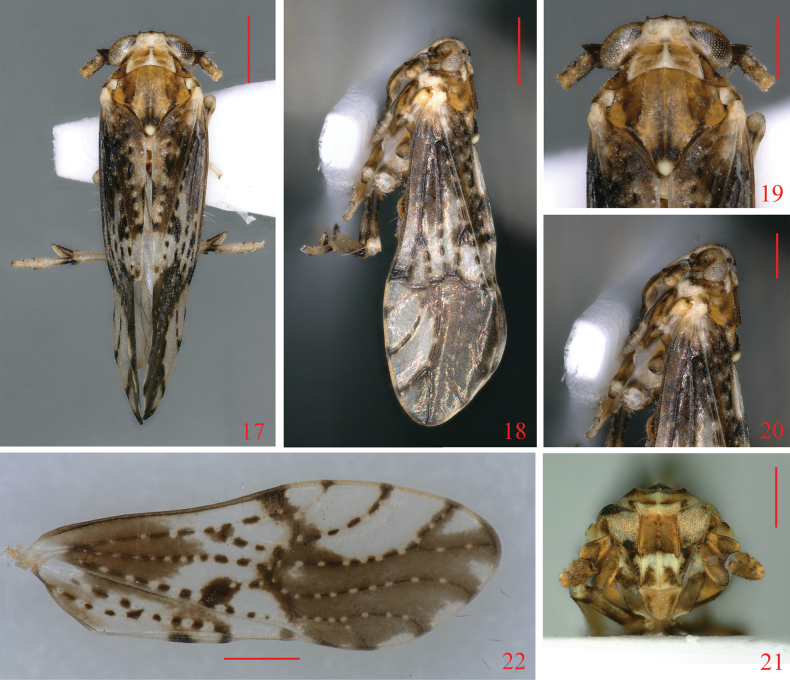
*Neobeloceraparvula* sp. nov. **17** male adult, dorsal view **18** same, lateral view **19** head and thorax, dorsal view **20** same, lateral view **21** face **22** forewing. Scale bars: 0.5 mm (**16, 17, 21**); 0.3 mm (**18–20**).

#### Description.

***Coloration*.** General coloration yellowish white to dark brown (Figs 17–22). Vertex (Fig. 19) yellowish white. Frons (Fig. 21) with basal half yellowish brown to brown, apical half yellowish white, with two triangular markings at apex. Genae (Fig. 21) dark brown, except longitudinal stripes below level of lower margin of ocelli yellowish white. Clypeus (Fig. 21) yellowish brown, except longitudinal stripes near lateral margin dark brown. Eyes (Figs 19–21) yellowish white to dark brown, ocelli (Fig. 20) reddish brown. Antennae (Figs 19–21) yellow to dark brown. Pronotum (Fig. 19) yellowish white to dark brown, median carina yellowish white. Mesonotum (Fig. 19) yellowish brown to dark brown, apex of scutellum yellowish white. Legs (Figs 17, 18) yellowish white, with dark brown maculations. Forewing (Fig. 22) light yellowish white, basal part near costal margin with large infuscate markings, and apical part from transverse veins to apex with dark-brown markings, veins with white spots at intervals. Wings hyaline with veins dark brown. Abdomen yellowish brown to dark brown.

**Figures 23–31.**
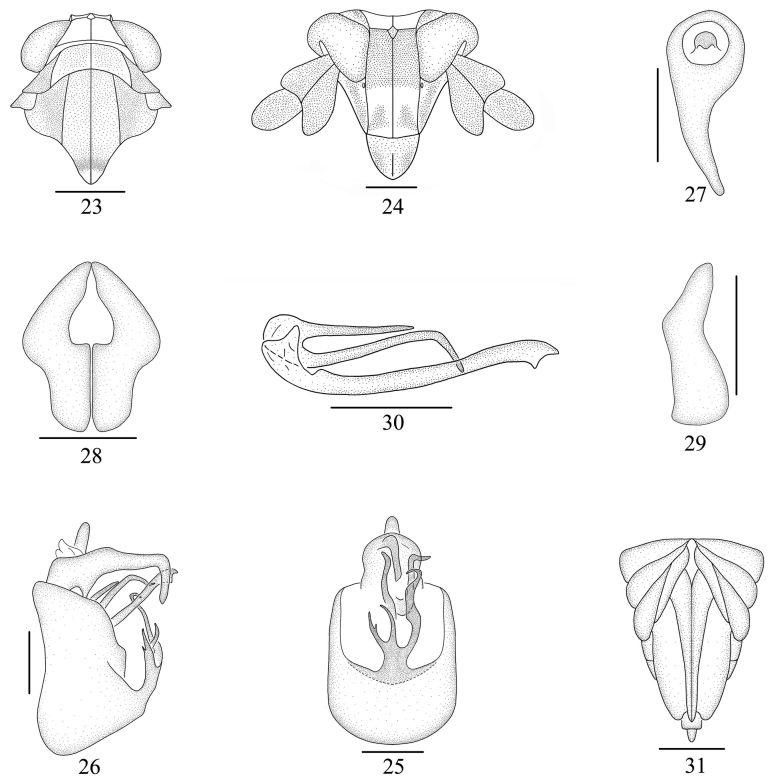
*Neobeloceraparvula* sp. nov. **23** head and thorax, dorsal view **24** face **25** male genitalia, posterior view **26** same, lateral view **27** anal segment, dorsal view **28** genital style, posterior view **29** same, lateral view **30** aedeagus, lateral view **31** female genitalia, posterior view. Scale bars: 0.5 mm (**31**); 0.2 mm (**23–27, 30**); 0.1 mm (**28, 29**).

***Head and thorax*.** Head including eyes slightly narrower than pronotum, in profile obtusely rounding into frons (Figs 19, 20). Vertex (Figs 19, 23) broad transversely, wider at base than long medially about 2.72: 1, width at apex narrower than at base about 1: 1.81, anterior margin produced medially, Y-shaped carina distinct. Frons (Figs 21, 24) in mid line longer than wide at widest part about 1.68: 1, widest above level of lower margin of eyes, median carina forked at extreme base. Postclypeus (Figs 21, 24) wider at base than frons at apex. Antennae (Figs 21, 24) reaching median part of postclypeus, basal segment shorter at midline than second segment about 1: 1.38, second segment long oval, somewhat compressed, longer than wide about 2.30: 1. Pronotum (Figs 19, 23) tricarinate, with anterior margin truncate, posterior margin incised strongly, lateral carinae running near anterolateral margin and reaching hind margin. Mesonotum (Figs 19, 23) tricarinate, longer in mid line than vertex and pronotum together about 2.03: 1, median carina reaching end of scutellum. Forewing (Fig. 22) broad and elongate, much longer than abdomen, longer in mid line than wide at widest part about 2.81: 1, predominately clear with distinctive white markings, wing apex acutely rounded; Sc, RA and RP unbranched; MP branched near wing apex, CuA 3-branched; junction of PCu + AA near midlength of clavus; fork of MP+CuA at near 2/3 length of clavus; fork RA+SC and RP near claval apex.

***Male genitalia*.** Anal segment (Figs 25–27) ring-like, ventral margin with a long process medially, bent ventrad medially. Pygofer (Figs 25, 26) in profile much longer ventrally than dorsally, in posterior view with opening longer than wide, ventral margin with medioventral process, three branched medially, right branch much longer than other two, sinuate, median branch nearly equal to left branch, left branch with a small process near apex. Genital styles (Figs 28, 29) stout, short, bent near middle, tapering apically. Aedeagus (Fig. 30) with phyllobase, phallus tubular, long, forked at apex, phyllobase slender, tubular, arising from base of aedeagus, two branched, longer branch bent ventrally near apex, shorter branch straight.

***Female genitalia*.** Female pygofer (Fig. 31) with gonocoxae narrower and long, basal angle sharply acute. Ovipositor slightly longer than pygofer. Gonangulum broad and large, apex round with medial margin concave, connected at base to gonapophyses and gonocoxae.

#### Host plant.

Bambusoideae.

#### Distribution.

China (Yunnan).

#### Remarks.

This new species is similar to *N.biprocessa* Li, Yang & Chen, 2020, but can be distinguished from the latter by the following features: (1) anal segment of male (Figs 25–27) with a long ventral process medially [anal segment without process in *N.biprocessa* (Li et al. 2020: fig. 2D)]; (2) pygofer (Fig. 25) with medioventral process [pygofer without medioventral process in *N.biprocessa* (Li et al. 2020: fig. 2D)]; and (3) genital styles (Figs 28, 29) stout and short [genital styles slender and long in *N.biprocessa* (Li et al. 2020: fig. 2H)].

## ﻿Discussion

**Figure 32.**
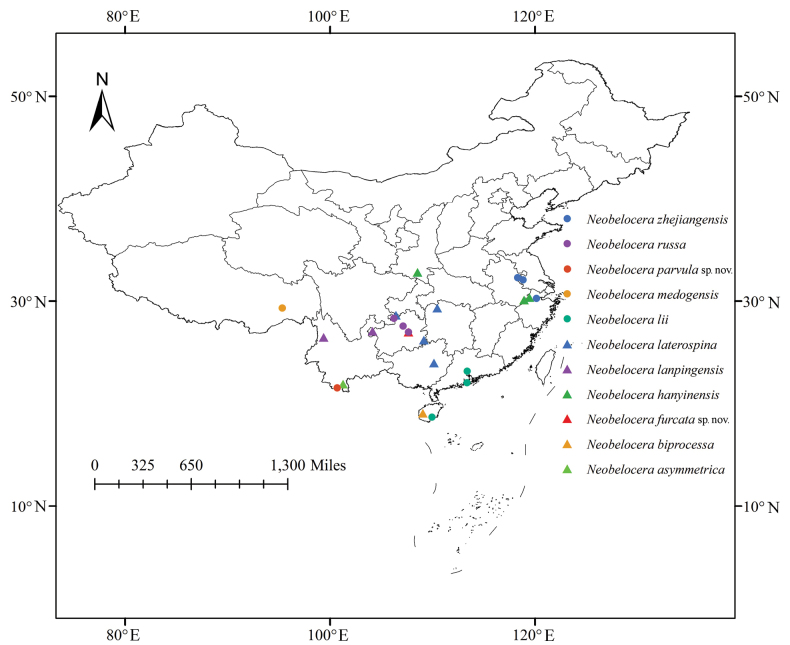
Distribution records of species of the genus *Neobelocera*.

Based on published data and our field surveys, the eleven described species within the genus *Neobelocera* are distributed in southern China (Fig. 32) in the Palaearctic region (Shaanxi) and the Oriental region (Guizhou, Yunnan, Hainan, Hunan, Guangxi, Guangdong, Chongqing, Zhejiang, Anhui, Jiangxi and Tibet). It seems that the genus is an endemic group of China. The complex and variable geomorphological environment and rich biological resources of the distribution area create a variety of habitat types, which are likely reasons for the rich species diversity of *Neobelocera*. We anticipate that additional species of *Neobelocera* will be found. Therefore, further investigation should be considered to fill the faunistic gaps, as it is obvious that many more taxa remain to be discovered and described.

Members of *Neobelocera* were found feeding exclusively on some native bamboos, with many specimens collected from the beginning of May to the end of September in Guizhou Province. So far, there are no collection records in other plants, which may suggest that the host of *Neobelocera* species are very limited.

## Supplementary Material

XML Treatment for Neobelocera

XML Treatment for Neobelocera furcata

XML Treatment for Neobelocera parvula

## ﻿Additional information

### Author ORCIDs

Hong-Xing Li https://orcid.org/0000-0002-6427-8875

### Data availability

All of the data that support the findings of this study are available in the main text.
